# High-Throughput Dispensing of Viscous Solutions for Biomedical Applications

**DOI:** 10.3390/mi13101730

**Published:** 2022-10-13

**Authors:** Richard A. Revia, Brandon Wagner, Matthew James, Miqin Zhang

**Affiliations:** 1 Department of Materials Science and Engineering, University of Washington, Seattle, WA 98195, USA; 2 Department of Neurological Surgery, University of Washington, Seattle, WA 98195, USA

**Keywords:** chitosan, alginate, cell scaffold, dispenser, high-throughput screening

## Abstract

Cells cultured in three-dimensional scaffolds express a phenotype closer to in vivo cells than cells cultured in two-dimensional containers. Natural polymers are suitable materials to make three-dimensional scaffolds to develop disease models for high-throughput drug screening owing to their excellent biocompatibility. However, natural polymer solutions have a range of viscosities, and none of the currently available liquid dispensers are capable of dispensing highly viscous polymer solutions. Here, we report the development of an automated scaffold dispensing system for rapid, reliable, and homogeneous creation of scaffolds in well-plate formats. We employ computer-controlled solenoid valves to regulate air pressure impinging upon a syringe barrel filled with scaffold solution to be dispensed. Automated dispensing of scaffold solution is achieved via a programmable software interface that coordinates solution extrusion and the movement of a dispensing head. We show that our pneumatically actuated dispensing system can evenly distribute high-viscosity, chitosan-based polymer solutions into 96- and 384-well plates to yield highly uniform three-dimensional scaffolds after lyophilization. We provide a proof-of-concept demonstration of high-throughput drug screening by culturing glioblastoma cells in scaffolds and exposing them to temozolomide. This work introduces a device that can hasten the creation of three-dimensional cell scaffolds and their application to high-throughput testing.

## 1. Introduction

In research investigations, biological cells are typically cultured in plastic Petri dishes or flasks, a technique that forces cells to grow upon a rigid, two-dimensional (2D) surface. 2D cell culture methods do not always yield cells that exhibit behavior similar to their counterpart cells living in native environments [1,2,3,4,5]. When cells cultured for study do not recapitulate their in vivo phenotypes, results obtained from those cultured cells exhibit little to no predictability and are often misleading, while animal models are expensive, time consuming, and present ethical dilemmas [6]. To grow cells that express a more physiologically relevant phenotype, three-dimensional (3D) scaffolds have been developed to provide cells with an environment that mimics what the cells would observe in vivo. In contrast to 2D cell culture techniques, 3D cell scaffolds may be engineered to display properties that are similar to the extracellular matrices (ECMs) preferred by cells [7,8,9,10,11]. For example, chitosan and alginate, two natural, biocompatible materials were combined in solution to form a polyelectrolyte complex followed by lyophilization to generate a 3D porous chitosan-alginate (CA) scaffold [12,13]. These polymers share a structure similar to that of glycosaminoglycans which are essential elements of some ECMs [14,15]. CA scaffolds have been used for promoting cell penetration, cellular function, and tissue growth, and for culturing cancer cells that yielded a more tumorigenic phenotype in comparison to those cultured in 2D [16,17]. Similarly, a biodegradable and biocompatible polymer, hyaluronic acid (HA), is one of the major glycosaminoglycan (GAG) components of the ECM found in the brain [18,19]. A chitosan-HA (CHA) scaffold was synthesized in previous studies to mimic a glioblastoma tumor microenvironment to yield cultured brain tumor cells [20].

While progress has been made as a result of the use of 3D scaffolds, a significant bottleneck exists in the production of 3D cell scaffolds. With respect to the large-scale techniques of scaffold synthesis, which include particulate leaching [21,22,23,24], gas foaming [25,26,27], thermally induced phase separation [16,28,29], and emulsion freeze drying [30,31], a general procedure for scaffold production is as follows: (1) creation of solution by dissolving a polymer in an appropriate solvent, (2) casting the polymer solution into a mold (typically the wells of a microplate), and (3) removal of the solvent through evaporation or sublimation to create so-called aerogels where the liquid phase of the solution is replaced by air [32,33]. The key chokepoint in this process is step two, wherein a polymer solution is cast into a mold by hand. While a large infrastructure exists for conducting high-throughput biological assays using 2D cell culture systems and microplates [34], the currently existing apparatuses of this infrastructure cannot handle solutions of such high viscosities as are requisite of many of the polymer solutions that form 3D scaffolds. On the other hand, pneumatically actuated dispensing systems are often employed in the electronics and packaging industries to distribute solder pastes and high-viscosity glues, but these systems are costly and do not meet the needs of researchers requiring full control of dispensing parameters, nor are they designed to comport with the dimensions of microplates.

Here, we report a scaffold-solution dispensing platform that can quickly deposit high-viscosity biomaterial solutions into microplates for generation of large quantities of 3D scaffolds ready for testing in high-throughput assays. We show that our pneumatically actuated dispensing system, built with inexpensive off-the-shelf components and a customized software interface, can easily dispense CA and CHA solutions of 2, 4, and 8 wt % into both 96- and 384-well microplates. The developed scaffolds are characterized by scanning electron microscopy (SEM) and pore size analysis. Further, we provide a proof-of-concept drug screening experiment by exposing glioma cells seeded onto scaffolds to the chemotherapeutic drug temozolomide (TMZ). We envision this scaffold dispensing device to lead to quicker discovery of drugs in vitro that have a more successful rate of translation to the clinic than drugs evaluated in 2D cell culture systems.

## 2. Materials and Methods

### 2.1. Materials

All chemicals were purchased from Sigma–Aldrich (St. Louis, MO, USA) unless otherwise specified. Chitosan (85% deacetylated, medium molecular weight), alginic acid sodium salt (from brown seaweed, MW = 80–120 kDa), and hyaluronic acid sodium salt (from *Streptococcus equi*) were used as received. Dulbecco’s modified Eagle media (DMEM), penicillin streptomycin (Pen Strep), and Dulbecco’s phosphate-buffered saline (DPBS) were purchased from Gibco (Gaithersburg, MD, USA). AlamarBlue reagent was purchased from Invitrogen (Carlsbad, CA, USA). Fetal bovine serum (FBS) was purchased from Atlanta Biologicals (Atlanta, GA, USA).

### 2.2. Scaffold Dispenser Components and Fabrication

A commercial 3D printer (P802NA, Shenzhen Zonestar Innovation Technology Co, Shenzhen, China) was cannibalized and modified by removing its 3D filament extruder and replacing it with a custom-made pneumatic scaffold dispensing nozzle. The pneumatic scaffold dispensing system consisted of an air pressure supply (provided through the laboratory gas infrastructure), a pressure regulator (R25-02b, Parker Watts, Cleveland, OH, USA), two 12 V DC solenoid valves (P0558, BACOENG), a solenoid control circuit, a syringe barrel holder (custom-made from quarter-inch thick clear acrylic sheets), and an 80 W heating pad (SHS0024, Tempco, Wood Dale, IL, USA) controlled by a thermostat (HJ Garden XH-W3002). A 12 V DC supply (PMT-12V150W1aa, Delta Electronics, Taipei, Taiwan) provided power to the solenoids. Control of the solenoids was achieved using low-voltage DC signals provided by an ATmega2560 MCU (Microchip Technologies, Chandler, AZ, USA) to bias an n-channel MOSFET (IRF630, STMicroelectronics, Geneva, Switzerland) that acts as a switch. A flyback diode (1N5400RLG, ON Semiconductor, Phoenix, AZ, USA) was placed in parallel with each solenoid.

To position each well of the microplate to be filled underneath the scaffold dispensing nozzle, a GUI was developed in the Python programming language (Python Software Foundation, Wilmington, DE, USA). The GUI program controlled both the 3D printer’s stepper motors and the scaffold dispenser’s solenoid valves to move the microplate and provide pressure to drive out the scaffold solution through a nozzle.

### 2.3. Preparation of CA and CHA Scaffold Solutions

Three CA scaffold solutions (2, 4, and 8 wt %) were prepared by slowly dissolving 2, 4, and 8 g of alginic acid sodium salt in 199 g of deionized water. The solution was mixed in a planetary centrifugal mixer (Thinky ARM-300, Thinky USA, Laguna Hills, CA, USA) at 2000 rpm for 3 min to dissolve residual clumps of polymer. Chitosan powder (2, 4, and 8 g) was introduced to the solution once the alginic acid sodium salt was fully dissolved. The CA solution was mixed again in the planetary centrifugal mixer at 2000 rpm for 3 min to evenly distribute the chitosan powder within the solution. Acetic acid was added dropwise to make a 1 wt % acetic acid solution. The solution was mixed in the planetary centrifugal mixer at 2000 rpm for 5 min. After dissolution of the chitosan powder, the polymer mixture was blended twice for 5 min to homogenize the polymer solution. The mixture was cooled in an ice bath after the blending steps to remove excess heat from within the solution.

Three CHA solutions (2, 4, and 8 wt %) were prepared by separately dissolving 2, 4, and 8 g of chitosan powder and 1 g of HA sodium salt in a 1 wt % acetic acid solution. Both solutions were left overnight at room temperature to ensure dissolution of the polymer. Upon dissolution, the two mixtures were combined and placed in a planetary centrifugal mixer (Thinky ARM-300, Thinky USA, Laguna Hills, CA, USA) at 2000 rpm for 5 min before blending to homogenize the polymer solution. The CHA solution was blended twice for 5 min and cooled in an ice bath for 10 min in between blending steps.

### 2.4. Scaffold Dispensing and Processing

CA and CHA scaffold solutions were cast into 96- or 384-well microplates via the automated scaffold dispenser. Scaffold solutions were loaded into a 60 mL syringe barrel (309654, BD, Franklin Lake, NJ, USA). The syringes were fitted with precision, 20 gauge, Luer-locking dispensing tips (6699A4, McMaster-Carr, Los Angeles, CA, USA). The syringe barrel was loaded into the scaffold dispensing device. The air pressure applied to the syringe barrel was adjusted using a pressure regulator; the exact applied pressure (60 kPa to 500 kPa) was uniquely adjusted for different percentages of scaffold (2, 4, and 8 wt % polymer) to achieve 2 mm of scaffold in each well. After dispensing each well with scaffold solution, microplates were centrifuged (Srovall Legend XT, Thermo Scientific, Waltham, MA, USA) at 1500 rpm for 1 min to degas air bubbles and placed in a freezer overnight at −20 °C for 24 h. Frozen scaffolds were lyophilized in a Labconco 6 freeze dryer for 1 to 3 days.

### 2.5. Scanning Electron Microscopy

SEM images were acquired using an FEI Sirion XL830 Dual Beam FIB/SEM (FEI Company, Hillsboro, OR). Lyophilized specimens were cut in half, mounted on aluminum pin stubs (16111, Ted Pella Inc., Redding, CA, USA) with carbon tape, and sputter coated with Au/Pd for 1 min at 18 mA before imaging. Images were taken with a 5 kV accelerating voltage, a spot size of 2, and 500× magnification.

### 2.6. Macroscopic Imaging

Macroscopic images were acquired using a stereotactic microscope (AmScope, Irvine, CA, USA). The lyophilized scaffolds were removed from their respective wells and imaged on their sides.

### 2.7. Scaffold Pore Area Analysis

The areas of the pores composing each scaffold were measured from representative SEM images using ImageJ software. Freehand selections were manually drawn around the perimeters of pores, and at least 60 pores per scaffold were analyzed to determine a median pore area for each scaffold. The distribution of pore areas within a scaffold represented as mean plus or minus the standard deviation.

### 2.8. Evaluation of Scaffold Solution Viscosity

The rheological properties of 4 wt % CA and CHA polymer solutions were measured with a stress-controlled rheometer (MCR 301, Anton Paar, Ostfildern, Germany). A parallel-plate geometry was used with a plate diameter of 25 mm. Viscosity was measured at a constant shear rate of 1 s^−1^ with a zero-stop gap of 1 mm at a constant temperature of 25 °C. Measurements were taken in 10 s intervals for 200 s.

### 2.9. Cell Culture

U-118 MG human glioblastoma cells were purchased from the American Type Culture Collection (ATCC, Manassas, VA). Cells were maintained according to the supplier’s protocol in fully supplemented DMEM with 10% FBS and 1% Pen Strep in a humidified incubator with 5% CO_2_ at 37 °C.

### 2.10. In Vitro Drug Response Analysis

U-118 MG cells (10,000 cells/well) were cultured in 4 wt % CA scaffolds cast in 384-well microplates. Cells were treated with TMZ three days after cell seeding on scaffolds. Drug concentrations used for the trials were 156, 312, 625, 1250, 2500, and 5000 µM with n = 12 wells per condition. Cell viability was investigated 3 days post-treatment with the alamarBlue assay. Briefly, 50 µL of alamarBlue solution (10% alamarBlue reagent in fully supplemented DMEM) was added to each well. Samples were incubated at 37 °C for 2 h, then, the alamarBlue solution was transferred to an opaque, black 96-well plate for fluorescent signal measurements using a SpectraMax M5 microplate reader (Molecular Devices, Union City, CA, USA) at an excitation wavelength of 560 nm and a fluorescence emission read at 590 nm. Cell viability was reported as a percent of viable cells relative to control cells treated with DPBS. The drug response profile was estimated using nonlinear least square estimation of the three parameter Hill equation using the lmfit package in Python.

## 3. Results

### 3.1. Automated Scaffold Dispenser Design

**Figure 1:**
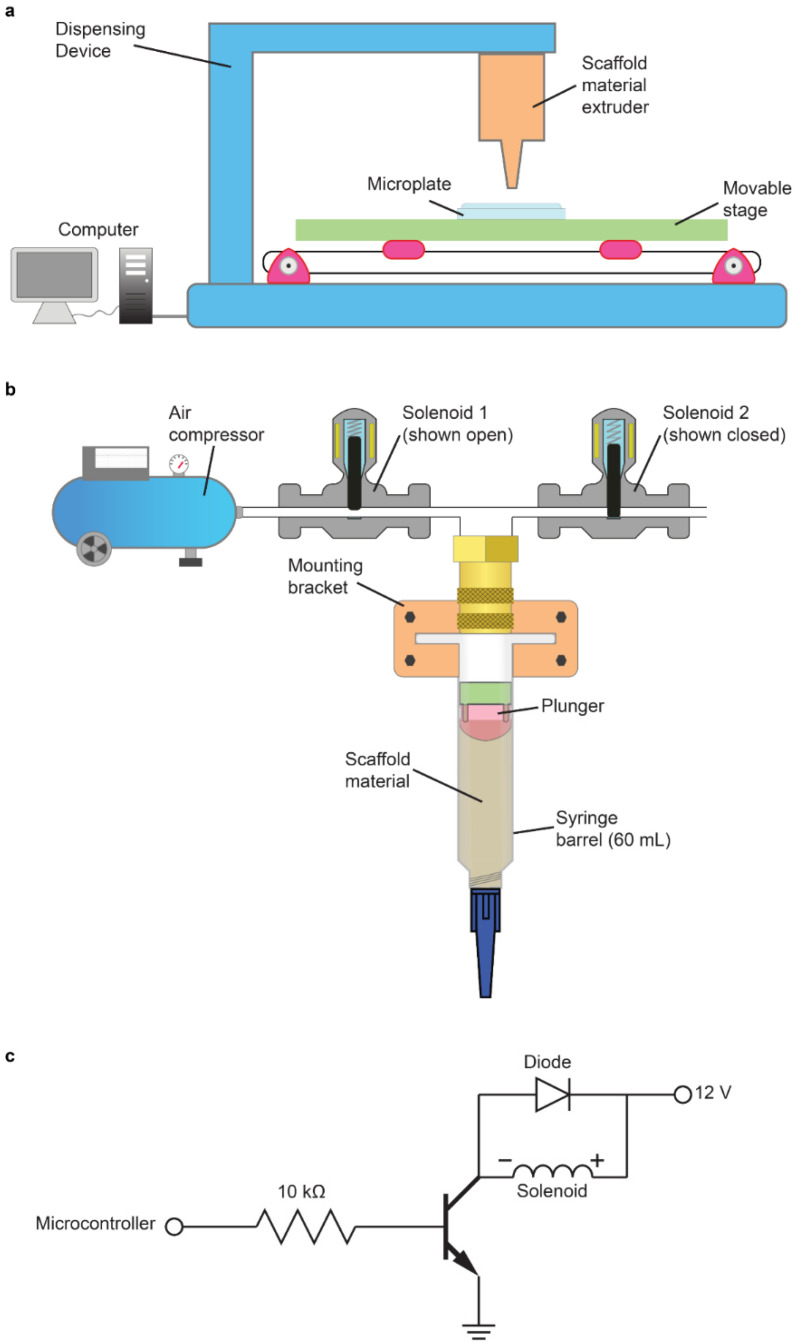
Automated scaffold dispenser design and illustration. (**a**) Automated scaffold dispensing apparatus based on a stepper-motor powered movable stage and a custom extruder capable of distributing high-viscosity materials. (**b**) Pneumatic extruder design detail. An external source of pressurized gas is used to depress the plunger of a syringe filled with scaffold solution; management of the applied pressure is achieved with a pressure regulator and two computer-controlled solenoid valves. (**c**) Solenoid valve control circuit.

The scaffold dispenser system consisted of a pneumatically actuated solution dispenser mounted to a commodity 3D printer as shown schematically in Figure 1a. Due to the high viscosities of the polymer solutions used to synthesize 3D scaffolds, any automated scaffold dispensing system requires a source of force strong enough to distribute the polymer solution from a large repository container to the miniature vessels of a microplate. Furthermore, the force applied to these high-viscosity solutions needs to be reliably and precisely controlled given the dimensions of the microplates involved in high-throughput drug screening. For instance, the individual wells of a 96-well microplate have a volume of 360 μL and a well diameter of 6.4 mm; similarly, the individual wells of a 384-well microplate have a volume of 120 μL and a well diameter of 3.65 mm. The small working areas of such microplates necessitate dispensing in an automated fashion with precision.

To generate short bursts of pressure with sufficient magnitude to press a viscous material through a small nozzle, we designed a pneumatically actuated fluid dispensing system as shown schematically in Figure 1b. This design consisted of two inexpensive solenoid valves, which are highly reliable fluid flow-control devices that may be readily actuated via computer-generated digital commands and an analog switching circuit (Figure 1c). These two solenoids modulated the pressure exerted on a 60 mL syringe barrel filled with scaffold material. A plunger was inserted into the syringe barrel above the scaffold material to aid uniform dispensing. The inlet of the syringe was connected to vinyl tubing that tees off a short distance above the opening of the barrel. One arm of the tee led to a solenoid valve (solenoid 1) that opened and closed to a source of pressurized air of about 200 kPa (although the optimal pressure for dispensing was dependent upon the scaffold solution viscosity). The other arm of the tee led to a second solenoid valve (solenoid two) that opened and closed to the atmosphere. When solenoid one was open while solenoid two was closed, as demonstrated in Figure 1b, the plunger was depressed by the pressurized air causing scaffold material to flow out of the tip of the syringe barrel. When solenoid one was closed while solenoid two was opened, the pressurized air source was cut off from reaching the top of the plunger and any excess pressure attempting to press down on the plunger found a pathway to the atmosphere through the opening represented by solenoid two. A computer program controlled the opening and closing of both solenoids automatically by instructing the hardware to apply current through them.

To achieve automated dispensing of an entire microplate, we cannibalized 3D printer parts for modification as shown schematically in Figure 1a. In place of the 3D printer’s filament extruder, we attached a syringe barrel loaded with scaffold solution. A microplate was placed upon the 3D printer’s stage and was situated underneath the scaffold material extruder. The movable stage was actuated by stepper motors which moved the microplate horizontally and vertically in precise step sizes so that one of the wells of the microplate was always directly beneath the tip of the scaffold material extruder when the stage was stationary. The same computer program that controlled the two solenoids was utilized to control the movement of the stepper motors and dictate the location of the microplate to consistently position the extruder above microplate wells.

### 3.2. Realized Scaffold Dispensing System

**Figure 2:**
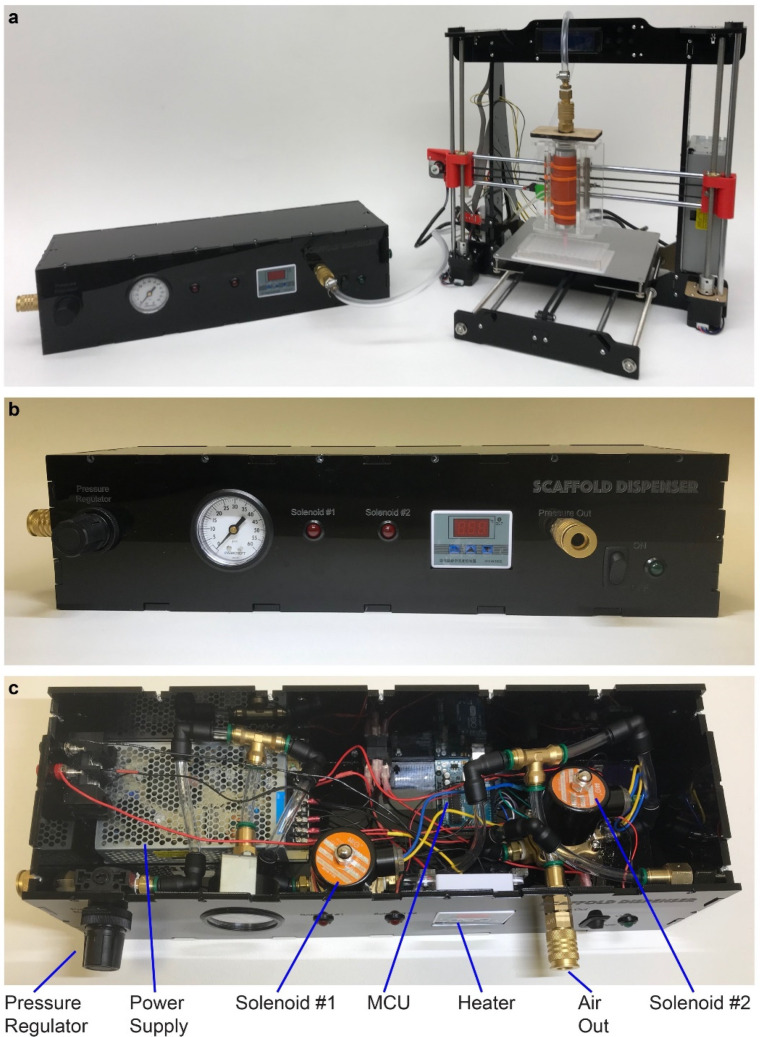
Realized scaffold dispenser. (**a**) Pneumatic housing (left) and modified 3D printer with syringe barrel (right). (**b**) Close-up of the pneumatic housing unit. (**c**) Inside the pneumatic housing unit with key components labelled. Pressurized gas is introduced to the pneumatic system through the quick connect fitting on the extreme left and is attached to the syringe barrel via the air out connection.

The actualized scaffold dispensing apparatus is shown in Figure 2a. An orange heating jacket surrounding the syringe barrel is shown in Figure 2a. The temperature of this 80 W heating pad was controlled via a thermostat residing in the pneumatic housing unit (Figure 2b,c). The heating pad was mounted to an aluminum tube, and a syringe barrel filled with scaffold solution was loaded inside the aluminum tube. When dispensing, the scaffold solution could be heated to a constant temperature in order to decrease the solution viscosity and facilitate distribution of high-viscosity fluids. The user could opt for nonheated dispensing when the fluid under study had low viscosity or if dispensing at medium viscosities. The amount of pressure applied during dispensing could be set using an adjustable pressure regulator.

**Figure 3:**
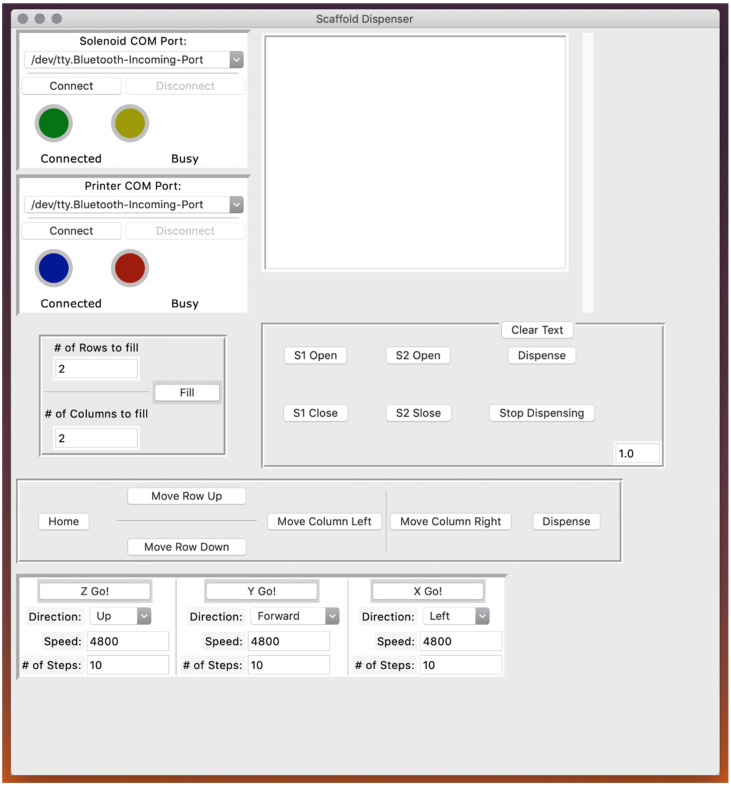
Graphical user interface. A screenshot of the scaffold dispensing control software.

Control of the scaffold dispensing system was realized through a custom-designed graphical user interface (GUI); a screenshot of the GUI is displayed in Figure 3. Through this program, a user can connect to the 3D printer to control the location of the microplate and scaffold extruder. If necessary, the user can separately connect to the microcontroller unit (MCU) that controls the solenoids. The user can select the type of well plate they wish to dispense into (e.g., 24, 48, 96, or 384). The number of steps the stepper motors required to translate the microplate the exact distance needed to position the scaffold extruder tip over the center of an adjacent well has been predetermined and hard coded into the software. Similarly, the user can adjust the dwell time, which is the amount of time solenoid one is open. Longer dwell times will apply air pressure to the scaffold solution for a greater duration. The appropriate amount of dwell time corresponds to the volume of the wells for a given microplate (i.e., larger well volume can accommodate more scaffold material, so pressure must be applied for a longer period of time in order to dispense more scaffold solution). Furthermore, the optimal dwell time is unique to a particular scaffold solution mixture, with higher viscosity polymer solutions typically requiring longer dwell times. However, the dwell time can be shortened by lowering the viscosity of the scaffold solution to be dispensed by increasing the temperature of the heating jacket surrounding the syringe barrel. Users may also input the number of rows and columns they wish to fill so that they can either fill each well of a microplate or just a small subset of wells.

### 3.3. High-Viscosity Scaffold Solutions

To demonstrate the ability of the scaffold dispenser to uniformly distribute scaffold materials into microplates, two exemplary polymer solutions were chosen. Chitosan and alginate are two widely used biocompatible materials that achieve excellent cell models when they are used as the substrate for cell culture [35,36,37,38,39]. Chitosan, a derivative of chitin, is a natural cationic polysaccharide derived from shrimp shells; alginate is a natural anionic polysaccharide derived from brown algae. Both materials have been shown to be biodegradable and induce a negligible immune response when exposed to living systems [40,41,42,43]. Additionally, both polymers share a structure that is similar to glycosaminoglycans, which are essential components of some ECMs. When used as a 3D cell scaffold, chitosan promotes cell adhesion, proliferation, and differentiation due to its hydrophilicity [44]. CA scaffolds were synthesized in previous studies for stem cell renewal due to their proxy structures of GAG and for tumor modeling due to their ability to mimic the tumor microenvironment and improve the tumorigenic potential of cultured cells [45,46,47,48]. Another biocompatible and biodegradable polymer is HA, a natural anionic polymer found in synovial fluid, cartilage, and skin; it is also one of the major glycosaminoglycan components in the brain’s ECM [49,50,51]. CHA scaffolds were synthesized in previous studies to serve as a mimic of the glioblastoma tumor microenvironment and to promote cartilage regeneration. Previous studies using chitosan-based 3D scaffolds for cell culture have yielded promising results that show the ability of these scaffolds to culture cancer cells that exhibit a more malignant and drug resistant phenotype compared to cancer cells cultured in Petri dishes [17,20].

**Figure 4:**
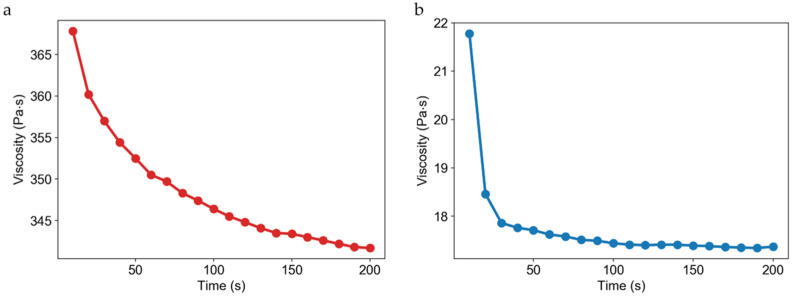
Viscosity of CHA and CA scaffolds. Rheological data of 4 wt % (**a**) CA and (**b**) CHA scaffold solutions. Viscosity is plotted against time showing both scaffold solutions have a shear thinning characteristic.

CA and CHA scaffolds were synthesized at polymer concentrations of 2, 4, and 8 wt %. Figure 4 displays rheological assessments of 4 wt % CA and CHA scaffolds performed at 25 °C. For reference, water has a viscosity of about 8.9 × 10^−4^ Pa·s at 25 °C, but 4 wt % CA and CHA exhibit a viscosity of at least four orders of magnitude greater at about 3.7 × 10^2^ Pa·s for CA and 2.1 × 10^1^ Pa·s for CHA. The large values of viscosity attendant to the solutions used to generate 3D scaffolds are why hand-casting is such a laborious and time-consuming process, and why specialized dispensing equipment is required for handling such solutions.

### 3.4. CA and CHA Scaffolds Automatically Dispensed into 96- and 384-Well Plates

**Figure 5:**
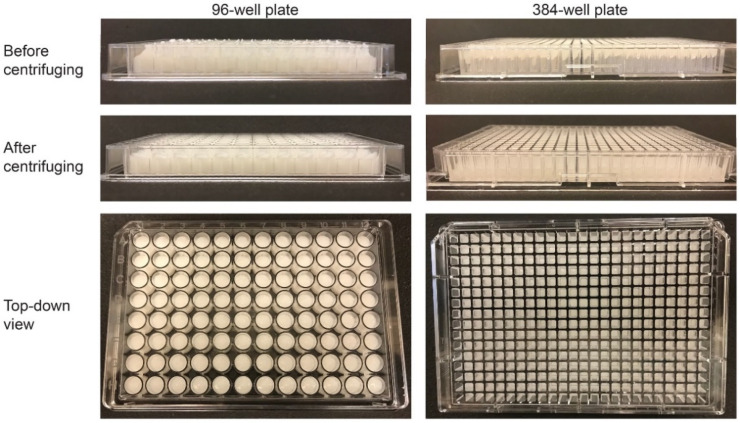
Dispensed scaffolds in 96- and 384-well plates. The images in the top row are side-on views of the microplates before they were centrifuged. A close inspection shows that the heights of the scaffolds were uneven among wells. The second row depicts side-on images of the scaffolds after centrifuging where the scaffolds heights were uniform among wells. The bottom row shows top-down views of fully populated 96- and 384-well microplates.

Figure 5 displays photographs of 2 wt % CA scaffolds dispensed in both a 96-well plate (left) and a 384-well plate (right). The dispensing time for this scaffold solution in the 96- and 384-well microplates was roughly 4 min and 15 min, respectively. Table 1 shows the various parameters used during this experiment. The top row of images in Figure 5 depicts the microplates immediately after automated dispensing. After dispensing, the height of the scaffold solution in the wells was not uniform; in order to address this issue, each microplate was centrifuged for 1 min at 1500 rpm immediately after dispensing to allow the scaffold solution to settle to the bottom of the well and form a homogeneous shape from well-to-well, as shown in the middle and bottom rows of Figure 5.

### 3.5. Characterization of Dispensed CA and CHA Scaffolds

**Figure 6:**
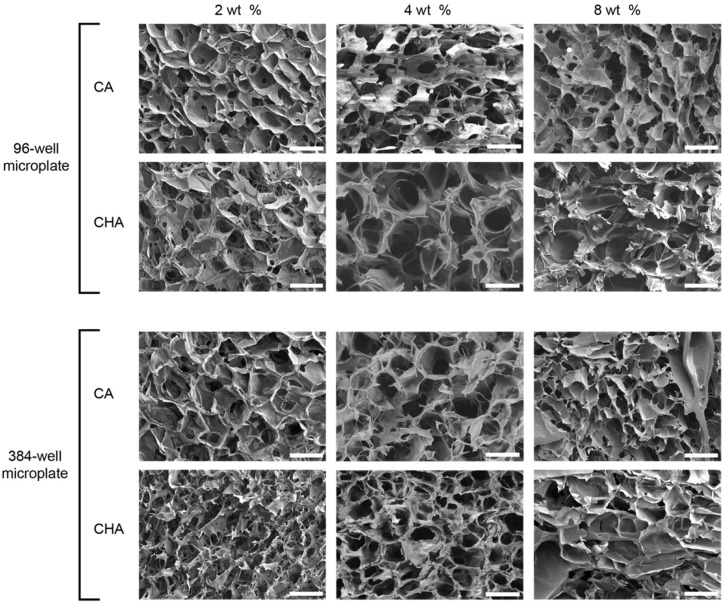
Evaluation of scaffold microstructure in both 96- and 384-well plates. Cross-sectional SEM images of CA and CHA scaffolds of 2, 4, and 8 wt % polymer cast in 96- and 384-well plates. Scale bars represent 100 µm.

**Figure 7:**
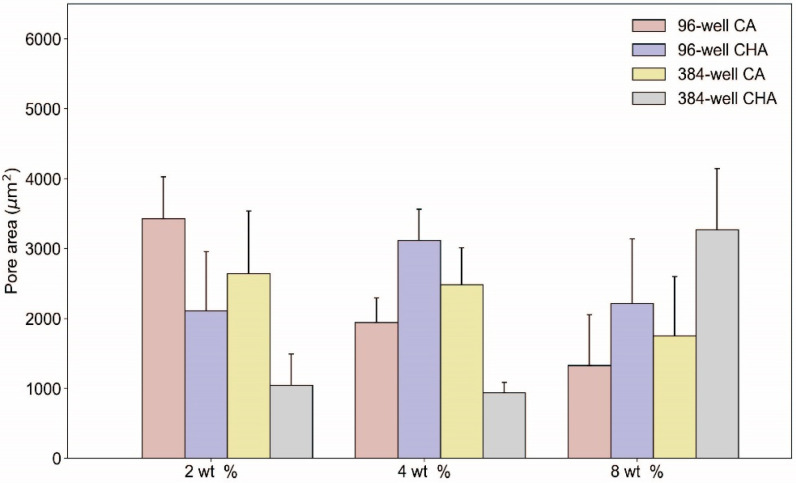
Pore area of CA and CHA scaffolds. The mean pore area of 2, 4, and 8 wt % CA and CHA scaffolds dispensed in both 96- and 384-well plates. Data are displayed as the average with the error bars indicating the standard deviation (*n* ≥ 60).

Dispensed and lyophilized CA and CHA scaffolds were analyzed by SEM to examine their pore size, morphology, interconnectivity, and uniformity. The main purpose of imaging was to confirm the existence of pores within the microstructure of the scaffolds. Scaffold porosity may be altered and optimized by tuning processing parameters such as solution viscosity, polymer concentrations, freezing rates, freezing temperatures, and acetic acid concentrations [52]. Despite a high degree of variability between the pore sizes of the scaffolds, as shown in the SEM images of Figure 6 and the pore size measurements reported in Figure 7, pores were successfully generated in 2, 4, and 8 wt % CA and CHA scaffolds dispensed in both 96- and 384-well microplates. Dispensing of the scaffold solutions was nontrivial because their viscosities change with varying shear rates (i.e., they are non-Newtonian fluids). Rheological measurements of 4 wt % CA and CHA solutions determined these polymer solutions to exhibit pseudoplastic thixotropic behavior where their viscosities decrease over time under a constant shear rate (Figure 4). Since viscosity is not independent of time when a constant force is applied, flow of the scaffold solution out of its container during dispensing was not uniform. However, the changing viscosity eventually reached a plateau during prolonged exposure to force. To reach this region of relatively constant viscosity, several “dummy” extrusions of scaffold were applied prior to dispensing into a microplate in order to obtain a scaffold solution of consistent viscosity, thus achieving a uniform volume of solutions dispensed in each well of a microplate.

The large amount of variability in pore size and morphology exhibited by the CA and CHA scaffolds herein (Figure 6 and Figure 7) may be attributed to insufficient blending during synthesis of scaffold solutions, resulting in a heterogeneous dispensing solution and subsequent creation of rough surfaces and nonuniform ice crystal growth during lyophilization. Additionally, the elongated pores evident in SEM images of a subset of the created scaffolds may be a result of adding more than 1 wt % of acetic acid to the scaffold solution, causing the solution viscosity to increase significantly and thereby result in a degradation of the desired pore structure. The large increase of solution viscosity due to higher amounts of acetic acid causes the diffusion rate of the scaffold polymer to decrease. When mobility of a polymer is low during crystallization and recrystallization of ice crystals, polymer dendrites cannot fully migrate to form the walls of the scaffold’s pores. Consequently, large irregular pores are formed when solution viscosity is high. Pore irregularity cannot be attributed to the scaffold dispensing technique itself because compressed air would only introduce gas bubbles within the solution, which is degassed during centrifugation.

**Figure 8:**
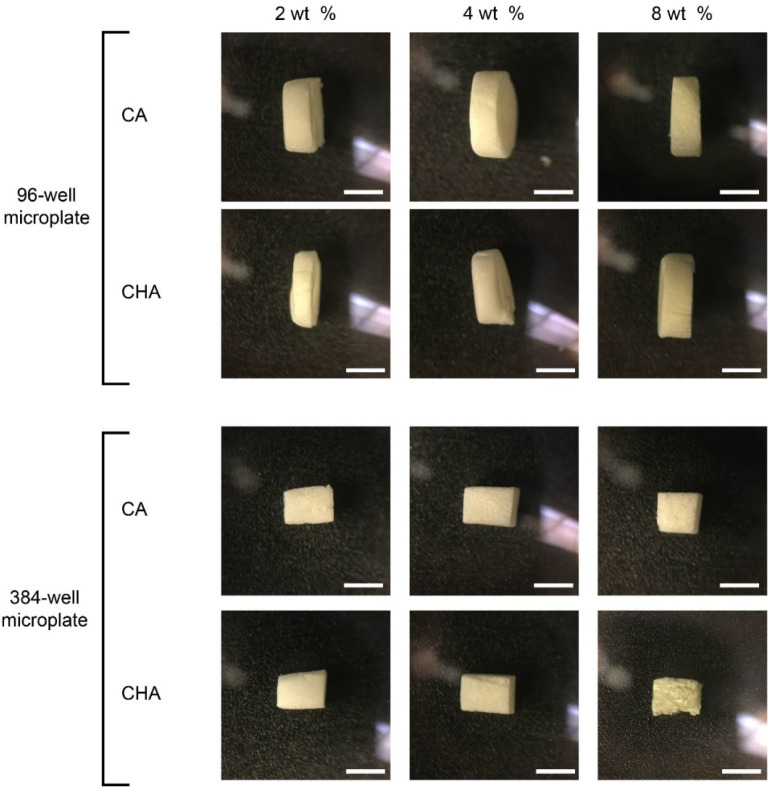
Photographs of scaffold sections cut from bulk scaffolds removed from both 96- and 384-well plates. Side view images of CA and CHA scaffolds of 2, 4, and 8 wt % polymer cast in 96- and 384-well plates. Scale bars represent 2 mm.

Figure 8 displays the photographs of the dispensed 3D scaffolds after being removed from the wells of their microplate reader containers and being hand cut into sections ~2 mm thick using a razor blade. These images show that the 3D scaffolds conform to the shape of their container and have an inherent supportive structure that allows them to retain the shape of the mold in which they were cast.

### 3.6. Drug Screening Proof-of-Concept

**Figure 9:**
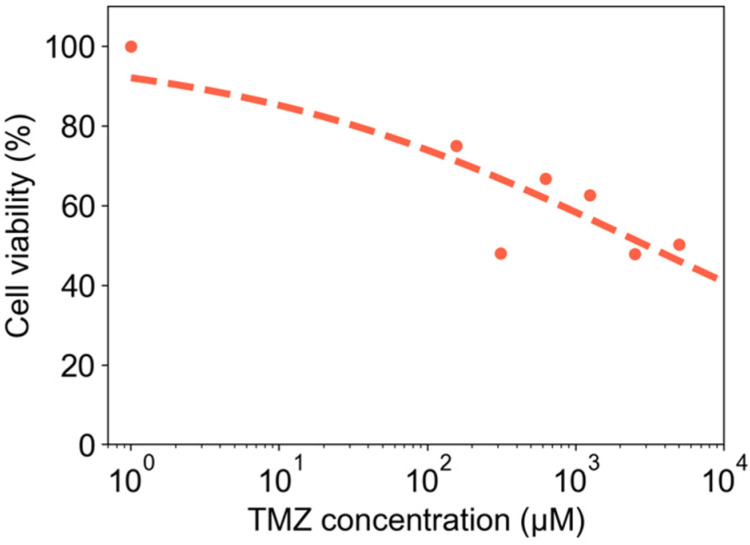
In vitro drug screening proof-of-concept. Dose-dependent cytotoxicity of TMZ on U-118 MG glioma cells cultured in 4 wt % CA scaffolds dispensed in a 384-well microplate. Cell viability was evaluated using the alamarBlue assay 3 days after treatment with TMZ. Data points (red circles) represent measured values whereas the dashed red line represents a best fit of the data to the Hill equation.

CA scaffolds (4 wt %) were dispensed in a 384-well microplate and used for in vitro drug screening to evaluate the cytotoxicity of the standard-of-care chemotherapeutic TMZ against glioma cells (U-118 MG). Cell viability of U-118 MG cells cultured on the CA scaffolds was measured as a function of TMZ concentration, and results are shown in Figure 9.

## 4. Discussion

Two-dimensional culture systems are a commonly used, convenient, and inexpensive commodities to grow and evaluate different types of cells in vitro for research endeavors; however, 2D culture systems do not provide stimuli that cells routinely experience in vivo, leading cells grown on 2D to not behave similarly to cells in vivo [53]. Three-dimensional culture systems, such as aerogels, provide an environment more comparable to what cells would experience if they were grown in vivo than 2D culture systems do. This allows cells grown in 3D to exhibit more realistic responses to external stimuli (e.g., pharmacotherapeutics, exogenous strains, and chemotactic agents) than those grown on 2D, and therefore serve as better models of disease for research studies. Therapeutic drug development is generally slow and expensive to undertake. In an effort to help with initial screening of drug libraries, a high-throughput 3D system is highly desirable as it allows us to test drug candidates against an accurate model of disease rather than weakened models that are less likely to produce therapies that will translate into clinical use. Our high-throughput dispensing system can easily dispense highly viscous polymer solutions, as we are able to fill 96-well plate and 384-well plate microplate readers with aerogels in less than 5 min and 20 min, respectively.

We sought to populate microplate readers with aerogels because microplate readers are widely used in the biomedical industry for cell culture and drug discovery, and many existing measurement devices capable of assaying cells in culture are based on the microplate reader format. We demonstrated the utility of dispensing aerogels into microplate readers for drug discovery with a proof-of-concept experiment wherein glioma cells cultured in 4 wt % CA scaffolds were exposed to the standard-of-care glioma pharmacologic agent TMZ. We showed an expected decreased in cell viability for increased TMZ concentration, thereby demonstrating that this system is amenable to high-throughput drug testing.

Other studies that describe dispensing of aerogel solutions for tissue engineering purposes typically focus on creating custom geometries to execute unique and highly specific experiments rather than for high-throughput studies. Tetik et al. created an aerogel dispenser using inkjet printing technology combined with lyophilization to generate 3D scaffolds with custom geometries [54]. Unlike our system, where the emphasis is on high-throughput dispensation into existing molds that are in common use for cell culture techniques, their system prioritizes single-use, tailor-made aerogel architectures. Both approaches have their merits, but our system is more suitable to existing drug-screening techniques and research studies requiring large sample sizes. Many other aerogel dispensing devices have been reported in the literature, where researchers have sought to create custom scaffold geometries rather than high quantities of rapidly dispensed aerogels [55,56,57,58,59,60].

Three-dimensional scaffolds are excellent at providing more realistic in vitro environments and stimuli for disease modeling. Chitosan and silk fibroin scaffolds were fabricated as 3D aerogels, and A549 lung cancer cells were shown to have a greater tendency to form spheres in these scaffolds which better mimicked human small-lung cell cancer than the same cells grown on 2D cell culture [61]. The cells were shown to have morphologies more similar to those found in vivo and a stronger resistance to doxorubicin (DOX). Poly(lactic-co-glycolic) acid and polycaprolactone (PCL) have been used to fabricate 3D scaffolds to evaluate ER-positive luminal A type T47D breast cancer cells and their response to the anticancer drug (Z)-4-Hydroxytamoxifen(4HT) [62]. Researchers found that the cells grown in 3D scaffolds were less sensitive to 4HT than those grown in 2D, and MDA-MB-231 breast cancer cells grown in 3D were more similar to cells found in vivo in terms of proliferation. There is an abundance of literature evaluating cells grown in 3D aerogels and their response to therapeutics, however, many of these studies only evaluate a few therapeutics or cell lines at certain time points. To achieve a high-throughput system to evaluate multiple therapeutics or cells lines, a robust and facile method to produce consistent scaffolds rapidly is needed. Therefore, we designed a device capable of dispensing both low-viscosity and high-viscosity solutions specifically for the creation of 3D scaffolds for cell culture. Results described herein show our device’s ability to dispense 2, 4, and 8 wt % CA aerogels at viscosities of 5.9 × 10^1^, 3.7 × 10^2^, and 6.6 × 10^2^ Pa·s, respectively. Our device was also able to dispense 2, 4, and 8 wt % CHA aerogels at viscosities of 3.7 × 10^−^^1^, 2.1 × 10^1^, and 1.3 × 10^2^ Pa·s, respectively. Although not described in this manuscript, we have also tested our system’s ability to dispense low-viscosity solutions such as water into microplates with control over the volume dispensed and speed of dispensing equivalent to or better than that shown with dispensing CA and CHA solutions. Likewise, we have been able to dispense other natural and synthetic polymers, not just CA and CHA, including gelatinized starch and PCL, which were used for pilot studies during the device’s design.

Our 3D scaffold dispensing platform around microplate readers can be utilized for biosensing applications. Biosensors are advantageous because they can provide real time, accurate measurements of critical parameters such as pH and dissolved oxygen (DO). Optical sensors used to measure pH and DO have been incorporated into the bottom of well plates in microplate readers to evaluate cell states in 2D, and a good correlation was found between proliferation and both pH and DO [63]; some minor modifications may be needed to apply to 3D models. Capacitance has also been used to correlate GFP-MCF-7 human breast cancer cells’ response to DOX treatment in 3D hydrogel scaffolds [64]. The capacitance was reduced after the therapeutic was administered, indicating the cell state can be monitored with this sensor. Silver nanoparticles and antibodies have also been incorporated in conjunction with PCL scaffolds as a separate capture substrate for surface enhanced Raman spectroscopy (SERS) evaluation of secreted proteins from adipose-derived mesenchymal stem cells [65], and the capture substrate was able to successfully detect various proteins, including differentiation markers. By incorporating biosensors with a high-throughput scaffold system, instantaneous monitoring of high-throughput drug screening assays is possible.

## 5. Conclusions

We developed an efficient and cost-effective scaffold dispensing system capable of quickly distributing high-viscosity solutions into miniature microplate formats with a high degree of uniformity in the resulting volume and 3D structure of the dispensed scaffolds from well to well. This device may be extended for use with viscous materials other than those intended to serve as porous scaffolds for 3D cell culture such as foods and glues, and this platform may also be used with larger well plate formats such as a 24-well plate. Precise control over the magnitude and dwell time of the air pressure applied to a scaffold solution held within a syringe using software commands facilitates reliable and rapid dispensing. Dispensing time was decreased to 4 min in 96-well microplates and 15 min for 384-well plates, compared to 25 min and 90 min required for hand casting hydrogels in 96-well and 384-well microplates, respectively. Expedited and automated scaffold dispensing may open new avenues for high-throughput assays that use 3D cell culture techniques in lieu of 2D platforms. A proof-of-concept experiment was performed in this study where glioma cells cultured in dispensed scaffolds residing in a 384-well microplate were exposed to the chemotherapeutic TMZ, and these microplates were used to rapidly assay cell death using a fluorescence-based evaluation method. With further incorporation of biosensors, instantaneous monitoring of high-throughput drug screening assays is possible. We envision that the 3D cell culture technique will help advance drug discovery by locating more efficacious and safer therapeutics, and that the high-throughput platform will accelerate clinical translation of potential drug candidates.

## Figures and Tables

**Table 1:** Parameters used for automated casting of CA and CHA scaffold material.

	CA						CHA					
Well Plate	96			384			96			384		
Weight Percent (%)	2	4	8	2	4	8	2	4	8	2	4	8
Pressure (kPa)	15	30	60	15	30	60	10	20	40	10	20	40
Dwell Time (ms)	120	120	120	120	120	120	100	100	100	100	100	100
Total Time (min)	4	4	4	15	15	15	4	4	4	15	15	15

## Data Availability

Not applicable.

## References

[1] Langhans S.A. (2018). Three-Dimensional in Vitro Cell Culture Models in Drug Discovery and Drug Repositioning. Front. Pharm..

[2] Wulftange W.J., Rose M.A., Garmendia-Cedillos M., da Silva D., Poprawski J.E., Srinivasachar D., Sullivan T., Lim L., Bliskovsky V.V., Hall M.D. (2019). Spatial Control of Oxygen Delivery to Three-dimensional Cultures Alters Cancer Cell Growth and Gene Expression. J. Cell Physiol..

[3] Sundarakrishnan A., Zukas H., Coburn J., Bertini B.T., Liu Z., Georgakoudi I., Baugh L., Dasgupta Q., Black L.D., Kaplan D.L. (2019). Bioengineered in Vitro Tissue Model of Fibroblast Activation for Modeling Pulmonary Fibrosis. ACS Biomater. Sci. Eng..

[4] Badekila A.K., Kini S., Jaiswal A.K. (2021). Fabrication Techniques of Biomimetic Scaffolds in Three-dimensional Cell Culture: A Review. J. Cell Physiol..

[5] Baker B.M., Chen C.S. (2012). Deconstructing the Third Dimension—How 3D Culture Microenvironments Alter Cellular Cues. J. Cell Sci..

[6] Arrowsmith J., Miller P. (2013). Trial Watch: Phase II and Phase III Attrition Rates 2011–2012. Nat. Rev. Drug Discov..

[7] Zhuravleva M., Gilazieva Z., Grigoriev T.E., Shepelev A.D., Tenchurin T.K., Kamyshinsky R., Krasheninnikov S.V., Orlov S., Caralogli G., Archipova S. (2019). In Vitro Assessment of Electrospun Polyamide-6 Scaffolds for Esophageal Tissue Engineering. J. Biomed. Mater. Res. B Appl. Biomater..

[8] Afewerki S., Sheikhi A., Kannan S., Ahadian S., Khademhosseini A. (2019). Gelatin-polysaccharide Composite Scaffolds for 3D Cell Culture and Tissue Engineering: Towards Natural Therapeutics. Bioeng. Transl. Med..

[9] Ma C., Chang B., Jing Y., Kim H., Liu X. (2018). Bio-Inspired Micropatterned Platforms Recapitulate 3D Physiological Morphologies of Bone and Dentinal Cells. Adv. Sci..

[10] Keirouz A., Chung M., Kwon J., Fortunato G., Radacsi N. (2020). 2D and 3D Electrospinning Technologies for the Fabrication of Nanofibrous Scaffolds for Skin Tissue Engineering: A Review. Wiley Interdiscip. Rev. Nanomed. Nanobiotechnol..

[11] Le M.N., Xu K., Wang Z., Beverung S., Steward R.L., Florczyk S.J. (2021). Evaluation of the Effect of 3D Porous Chitosan-alginate Scaffold Stiffness on Breast Cancer Proliferation and Migration. J. Biomed. Mater. Res. A.

[12] Kievit F.M., Florczyk S.J., Leung M.C., Wang K., Wu J.D., Silber J.R., Ellenbogen R.G., Lee J.S.H., Zhang M. (2014). Proliferation and Enrichment of CD133+ Glioblastoma Cancer Stem Cells on 3D Chitosan-Alginate Scaffolds. Biomaterials.

[13] Rouhollahi A., Ilegbusi O., Florczyk S., Xu K., Foroosh H. (2020). Effect of Mold Geometry on Pore Size in Freeze-Cast Chitosan-Alginate Scaffolds for Tissue Engineering. Ann. Biomed. Eng..

[14] Croisier F., Jérôme C. (2013). Chitosan-Based Biomaterials for Tissue Engineering. Eur. Polym. J..

[15] Tamimi M., Rajabi S., Pezeshki-Modaress M. (2020). Cardiac ECM/Chitosan/Alginate Ternary Scaffolds for Cardiac Tissue Engineering Application. Int. J. Biol. Macromol..

[16] Erickson A.E., Sun J., Lan Levengood S.K., Swanson S., Chang F.-C., Tsao C.T., Zhang M. (2019). Chitosan-Based Composite Bilayer Scaffold as an in Vitro Osteochondral Defect Regeneration Model. Biomed. Microdev..

[17] Wang K., Kievit F.M., Erickson A.E., Silber J.R., Ellenbogen R.G., Zhang M. (2016). Culture on 3D Chitosan-hyaluronic Acid Scaffolds Enhances Stem Cell Marker Expression and Drug Resistance in Human Glioblastoma Cancer Stem Cells. Adv. Healthc. Mater..

[18] Lau L.W., Cua R., Keough M.B., Haylock-Jacobs S., Yong V.W. (2013). Pathophysiology of the Brain Extracellular Matrix: A New Target for Remyelination. Nat. Rev. Neurosci..

[19] Entekhabi E., Haghbin Nazarpak M., Shafieian M., Mohammadi H., Firouzi M., Hassannejad Z. (2021). Fabrication and in Vitro Evaluation of 3D Composite Scaffold Based on Collagen/Hyaluronic Acid Sponge and Electrospun Polycaprolactone Nanofibers for Peripheral Nerve Regeneration. J. Biomed. Mater. Res. A.

[20] Erickson A.E., Lan Levengood S.K., Sun J., Chang F., Zhang M. (2018). Fabrication and Characterization of Chitosan–Hyaluronic Acid Scaffolds with Varying Stiffness for Glioblastoma Cell Culture. Adv. Healthc. Mater..

[21] Hutmacher D.W. (2001). Scaffold Design and Fabrication Technologies for Engineering Tissues—State of the Art and Future Perspectives. J. Biomater. Sci. Polym. Ed..

[22] Li L., Shi X., Wang Z., Guo M., Wang Y., Jiao Z., Zhang P. (2019). Porous Scaffolds of Poly (Lactic-Co-Glycolic Acid) and Mesoporous Hydroxyapatite Surface Modified by Poly (γ-Benzyl-l-Glutamate)(PBLG) for in Vivo Bone Repair. ACS Biomater. Sci. Eng..

[23] Sola A., Bertacchini J., D’Avella D., Anselmi L., Maraldi T., Marmiroli S., Messori M. (2019). Development of Solvent-Casting Particulate Leaching (SCPL) Polymer Scaffolds as Improved Three-Dimensional Supports to Mimic the Bone Marrow Niche. Mater. Sci. Eng. C.

[24] Murugan S., Parcha S.R. (2021). Fabrication Techniques Involved in Developing the Composite Scaffolds PCL/HA Nanoparticles for Bone Tissue Engineering Applications. J. Mater. Sci. Mater. Med..

[25] Rao F., Yuan Z., Li M., Yu F., Fang X., Jiang B., Wen Y., Zhang P. (2019). Expanded 3D Nanofibre Sponge Scaffolds by Gas-Foaming Technique Enhance Peripheral Nerve Regeneration. Artif. Cells Nanomed. Biotechnol..

[26] Dattola E., Parrotta E.I., Scalise S., Perozziello G., Limongi T., Candeloro P., Coluccio M.L., Maletta C., Bruno L., de Angelis M.T. (2019). Development of 3D PVA Scaffolds for Cardiac Tissue Engineering and Cell Screening Applications. RSC Adv..

[27] Yuan Z., Li Y., Zhao D., Zhang K., Wang F., Wang C., Wen Y. (2018). High Efficiency 3D Nanofiber Sponge for Bilirubin Removal Used in Hemoperfusion. Colloids Surf. B Biointerfaces.

[28] Conoscenti G., Carfì Pavia F., Ongaro A., Brucato V., Goegele C., Schwarz S., Boccaccini A.R., Stoelzel K., la Carrubba V., Schulze-Tanzil G. (2019). Human Nasoseptal Chondrocytes Maintain Their Differentiated Phenotype on PLLA Scaffolds Produced by Thermally Induced Phase Separation and Supplemented with Bioactive Glass 1393. Connect. Tissue Res..

[29] Rad R.M., Atila D., Akgün E.E., Evis Z., Keskin D., Tezcaner A. (2019). Evaluation of Human Dental Pulp Stem Cells Behavior on a Novel Nanobiocomposite Scaffold Prepared for Regenerative Endodontics. Mater. Sci. Eng. C.

[30] Grenier J., Duval H., Barou F., Lv P., David B., Letourneur D. (2019). Mechanisms of Pore Formation in Hydrogel Scaffolds Textured by Freeze-Drying. Acta Biomater..

[31] Reyna-Urrutia V.A., Mata-Haro V., Cauich-Rodriguez J.V., Herrera-Kao W.A., Cervantes-Uc J.M. (2019). Effect of Two Crosslinking Methods on the Physicochemical and Biological Properties of the Collagen-Chitosan Scaffolds. Eur. Polym. J..

[32] Baldino L., Concilio S., Cardea S., Reverchon E. (2016). Interpenetration of Natural Polymer Aerogels by Supercritical Drying. Polymers.

[33] Yahya E.B., Amirul A.A., HPS A.K., Olaiya N.G., Iqbal M.O., Jummaat F., AK A.S., Adnan A.S. (2021). Insights into the Role of Biopolymer Aerogel Scaffolds in Tissue Engineering and Regenerative Medicine. Polymers.

[34] Worthington P., Drake K.M., Li Z., Napper A.D., Pochan D.J., Langhans S.A. (2019). Implementation of a High-Throughput Pilot Screen in Peptide Hydrogel-Based Three-Dimensional Cell Cultures. SLAS DISCOVERY Adv. Life Sci. R D.

[35] Li Z., Ramay H.R., Hauch K.D., Xiao D., Zhang M. (2005). Chitosan-Alginate Hybrid Scaffolds for Bone Tissue Engineering. Biomaterials.

[36] Schulz A., Rickmann A., Wahl S., Germann A., Stanzel B.V., Januschowski K., Szurman P. (2020). Alginate-and Hyaluronic Acid–Based Hydrogels as Vitreous Substitutes: An in Vitro Evaluation. Transl. Vis. Sci. Technol..

[37] Chang P.-H., Chao H.-M., Chern E., Hsu S. (2021). Chitosan 3D Cell Culture System Promotes Naïve-like Features of Human Induced Pluripotent Stem Cells: A Novel Tool to Sustain Pluripotency and Facilitate Differentiation. Biomaterials.

[38] Su X., Chen L., Han S., Niu G., Ren J., Ke C. (2020). Preparation and Characterization of a Novel Triple Composite Scaffold Containing Silk Fiborin, Chitosan, and Alginate for 3D Culture of Colonic Carcinoma Cells In Vitro. Med. Sci. Monit..

[39] Zhao X., Hu D.A., Wu D., He F., Wang H., Huang L., Shi D., Liu Q., Ni N., Pakvasa M. (2021). Applications of Biocompatible Scaffold Materials in Stem Cell-Based Cartilage Tissue Engineering. Front. Bioeng. Biotechnol..

[40] Hutmacher D.W., Goh J.C.H., Teoh S.H. (2001). An Introduction to Biodegradable Materials for Tissue Engineering Applications. Ann. Acad. Med. Singap..

[41] Suh J.-K.F., Matthew H.W.T. (2000). Application of Chitosan-Based Polysaccharide Biomaterials in Cartilage Tissue Engineering: A Review. Biomaterials.

[42] Eslahi M., Dana P.M., Asemi Z., Hallajzadeh J., Mansournia M.A., Yousefi B. (2021). The Effects of Chitosan-Based Materials on Glioma: Recent Advances in Its Applications for Diagnosis and Treatment. Int. J. Biol. Macromol..

[43] Reig-Vano B., Tylkowski B., Montané X., Giamberini M. (2021). Alginate-Based Hydrogels for Cancer Therapy and Research. Int. J. Biol. Macromol..

[44] Levengood S.K.L., Zhang M. (2014). Chitosan-Based Scaffolds for Bone Tissue Engineering. J. Mater. Chem. B.

[45] Florczyk S.J., Kievit F.M., Wang K., Erickson A.E., Ellenbogen R.G., Zhang M. (2016). 3D Porous Chitosan-Alginate Scaffolds Promote Proliferation and Enrichment of Cancer Stem-like Cells. J. Mater. Chem. B.

[46] Kievit F.M., Florczyk S.J., Leung M.C., Veiseh O., Park J.O., Disis M.L., Zhang M. (2010). Chitosan–Alginate 3D Scaffolds as a Mimic of the Glioma Tumor Microenvironment. Biomaterials.

[47] Li Z., Leung M., Hopper R., Ellenbogen R., Zhang M. (2010). Feeder-Free Self-Renewal of Human Embryonic Stem Cells in 3D Porous Natural Polymer Scaffolds. Biomaterials.

[48] Rahmati M., Pennisi C.P., Mobasheri A., Mozafari M. (2018). Bioengineered Scaffolds for Stem Cell Applications in Tissue Engineering and Regenerative Medicine. Cell Biol. Transl. Med..

[49] Wang X., He J., Wang Y., Cui F.-Z. (2012). Hyaluronic Acid-Based Scaffold for Central Neural Tissue Engineering. Interface Focus.

[50] Correia C.R., Moreira-Teixeira L.S., Moroni L., Reis R.L., van Blitterswijk C.A., Karperien M., Mano J.F. (2011). Chitosan Scaffolds Containing Hyaluronic Acid for Cartilage Tissue Engineering. Tissue Eng. Part C Methods.

[51] Yoo H.S., Lee E.A., Yoon J.J., Park T.G. (2005). Hyaluronic Acid Modified Biodegradable Scaffolds for Cartilage Tissue Engineering. Biomaterials.

[52] Florczyk S.J., Kim D., Wood D.L., Zhang M. (2011). Influence of Processing Parameters on Pore Structure of 3D Porous Chitosan-Alginate Polyelectrolyte Complex Scaffolds. J. Biomed. Mater. Res. A.

[53] Duval K., Grover H., Han L.-H., Mou Y., Pegoraro A.F., Fredberg J., Chen Z. (2017). Modeling Physiological Events in 2D vs. 3D Cell Culture. Physiology.

[54] Tetik H., Yang G., Tan W., Fong A., Lei S., Weker J.N., Lin D. (2020). High Speed In-Situ X-Ray Imaging of 3D Freeze Printing of Aerogels. Addit. Manuf..

[55] Tetik H., Wang Y., Sun X., Cao D., Shah N., Zhu H., Qian F., Lin D. (2021). Additive Manufacturing of 3D Aerogels and Porous Scaffolds: A Review. Adv. Funct. Mater..

[56] Iglesias-Mejuto A., García-González C.A. (2021). 3D-Printed Alginate-Hydroxyapatite Aerogel Scaffolds for Bone Tissue Engineering. Mater. Sci. Eng. C.

[57] Lee H., Kim G. (2011). Cryogenically Fabricated Three-Dimensional Chitosan Scaffolds with Pore Size-Controlled Structures for Biomedical Applications. Carbohydr. Polym..

[58] Kim G., Ahn S., Yoon H., Kim Y., Chun W. (2009). A Cryogenic Direct-Plotting System for Fabrication of 3D Collagen Scaffolds for Tissue Engineering. J. Mater. Chem..

[59] Morris V.B., Nimbalkar S., Younesi M., McClellan P., Akkus O. (2017). Mechanical Properties, Cytocompatibility and Manufacturability of Chitosan: PEGDA Hybrid-Gel Scaffolds by Stereolithography. Ann. Biomed. Eng..

[60] Yan Y., Xiong Z., Hu Y., Wang S., Zhang R., Zhang C. (2003). Layered Manufacturing of Tissue Engineering Scaffolds via Multi-Nozzle Deposition. Mater. Lett..

[61] Li J., Zhou Y., Chen W., Yuan Z., You B., Liu Y., Yang S., Li F., Qu C., Zhang X. (2018). A Novel 3D in Vitro Tumor Model Based on Silk Fibroin/Chitosan Scaffolds to Mimic the Tumor Microenvironment. ACS Appl. Mater. Interfaces.

[62] Rijal G., Bathula C., Li W. (2017). Application of Synthetic Polymeric Scaffolds in Breast Cancer 3D Tissue Cultures and Animal Tumor Models. Int. J. Biomater..

[63] Naciri M., Kuystermans D., Al-Rubeai M. (2008). Monitoring PH and Dissolved Oxygen in Mammalian Cell Culture Using Optical Sensors. Cytotechnology.

[64] Lee S.-M., Han N., Lee R., Choi I.-H., Park Y.-B., Shin J.-S., Yoo K.-H. (2016). Real-Time Monitoring of 3D Cell Culture Using a 3D Capacitance Biosensor. Biosens. Bioelectron..

[65] Ko J., Ham J., Lee H., Lee K., Koh W.-G. (2021). Integration of a Fiber-Based Cell Culture and Biosensing System for Monitoring of Multiple Protein Markers Secreted from Stem Cells. Biosens. Bioelectron..

